# Comprehensive analysis of coding-lncRNA gene co-expression network uncovers conserved functional lncRNAs in zebrafish

**DOI:** 10.1186/s12864-018-4458-7

**Published:** 2018-05-09

**Authors:** Wen Chen, Xuan Zhang, Jing Li, Shulan Huang, Shuanglin Xiang, Xiang Hu, Changning Liu

**Affiliations:** 1 Key Laboratory of Tropical Plant Resources and Sustainable Use, Xishuangbanna Tropical Botanical Garden, Chinese Academy of Sciences, Kunming, China; 2 State Key Laboratory of Developmental Biology of Freshwater Fish, School of Life Sciences, Hunan Normal University, Changsha, China

**Keywords:** LncRNA, Zebrafish, Co-expression network, Gene ontology, KEGG, Conservation

## Abstract

**Background:**

Zebrafish is a full-developed model system for studying development processes and human disease. Recent studies of deep sequencing had discovered a large number of long non-coding RNAs (lncRNAs) in zebrafish. However, only few of them had been functionally characterized. Therefore, how to take advantage of the mature zebrafish system to deeply investigate the lncRNAs’ function and conservation is really intriguing.

**Results:**

We systematically collected and analyzed a series of zebrafish RNA-seq data, then combined them with resources from known database and literatures. As a result, we obtained by far the most complete dataset of zebrafish lncRNAs, containing 13,604 lncRNA genes (21,128 transcripts) in total. Based on that, a co-expression network upon zebrafish coding and lncRNA genes was constructed and analyzed, and used to predict the Gene Ontology (GO) and the KEGG annotation of lncRNA. Meanwhile, we made a conservation analysis on zebrafish lncRNA, identifying 1828 conserved zebrafish lncRNA genes (1890 transcripts) that have their putative mammalian orthologs. We also found that zebrafish lncRNAs play important roles in regulation of the development and function of nervous system; these conserved lncRNAs present a significant sequential and functional conservation, with their mammalian counterparts.

**Conclusions:**

By integrative data analysis and construction of coding-lncRNA gene co-expression network, we gained the most comprehensive dataset of zebrafish lncRNAs up to present, as well as their systematic annotations and comprehensive analyses on function and conservation. Our study provides a reliable zebrafish-based platform to deeply explore lncRNA function and mechanism, as well as the lncRNA commonality between zebrafish and human.

**Electronic supplementary material:**

The online version of this article (10.1186/s12864-018-4458-7) contains supplementary material, which is available to authorized users.

## Background

LncRNAs are termed as a heterogeneous class of transcripts with the length over 200 bp and without the potential of protein-coding [1–3]. By now, there are ten thousands of lncRNAs discovered across human, mouse, nematode, zebrafish etc. [4–6]. Meanwhile, an increasing number of studies demonstrated that lncRNAs have special functions in diverse biological processes and can extensively regulate gene expression and chromatin status, and thus influencing cellular homostasis [7–9]. As a result, these enormous amounts of lncRNAs can arrange to a complicated and fine-controlled regulation network for gene expression, mutation and dysregulation of them are likely linked to diverse human diseases and abnormal development [10–12].

Zebrafish as a powerful model for studying vertebrate biology has a relatively long history, on account of its clear development pattern and genetic background [13]. Large-scale genetic screens have identified hundreds of mutant phenotypes, many of which could imitate human disease status, providing a simple and accessible system for investigating the corresponding human diseases [14–16]. At the early stage of this kind of research, the focal spot is primarily on protein-coding gene. The well-characterized cases of using zebrafish as a model for studying protein roles in human disease are coming from the hematopoietic diseases, like ALAS2 in a microcytic, hypochromic anemia, UROD in porphyria, and etc. [17]. The semblable studies are also illuminated in Cardiovascular-, Kidney- and other organ- disorders [18, 19]. However, the similar studies of using the zebrafish as a model to probe lncRNAs’ function are still at infancy, because of the insufficient annotation and the lack of systematic survey.

To date, coupled with the progress of next generation sequencing in model organisms, it is feasible of large-scale discovering and annotating and integratively computational analyzing of zebrafish lncRNAs. By using chromatin marks, poly(A)-site mapping and RNA-Seq data, Ulitsky et.al had identified 550 distinct lncRNAs in zebrafish, with 29 lncRNAs having detectable sequence similarity with their putative mammalian orthologs and some having conserved genomic locations [20]. By a time-series of RNA-seq experiments, Andrea Pauli et al. had defined 1133 noncoding multi-exonic transcripts expressed at eight stages during early zebrafish embryogenesis, and found that zebrafish lncRNAs share many of the characteristics of their mammalian counterparts [21].

In this work, to better utilize the mature zebrafish system to uncover the commonality of lncRNAs’ function and conservation between zebrafish and human, and further provide a research platform of zebrafish model organisms to explore the function of lncRNAs in development processes and human disease, we presented and analyzed the most complete collection and annotation of zebrafish lncRNAs, by comprehensively integrating various data resources from public RNA-seq datasets, databases and literatures. By establishing a co-expression network upon lncRNA- and coding- genes, we set out to optimize the present gene annotation of zebrafish lncRNAs, to predict their potential functions, and to hunt their putative mammalian orthologs, so as to illuminate their functional and mechanism conservation between species.

## Results

### Comprehensive collection and integrative data analysis of zebrafish lncRNAs

**Fig. 1:**
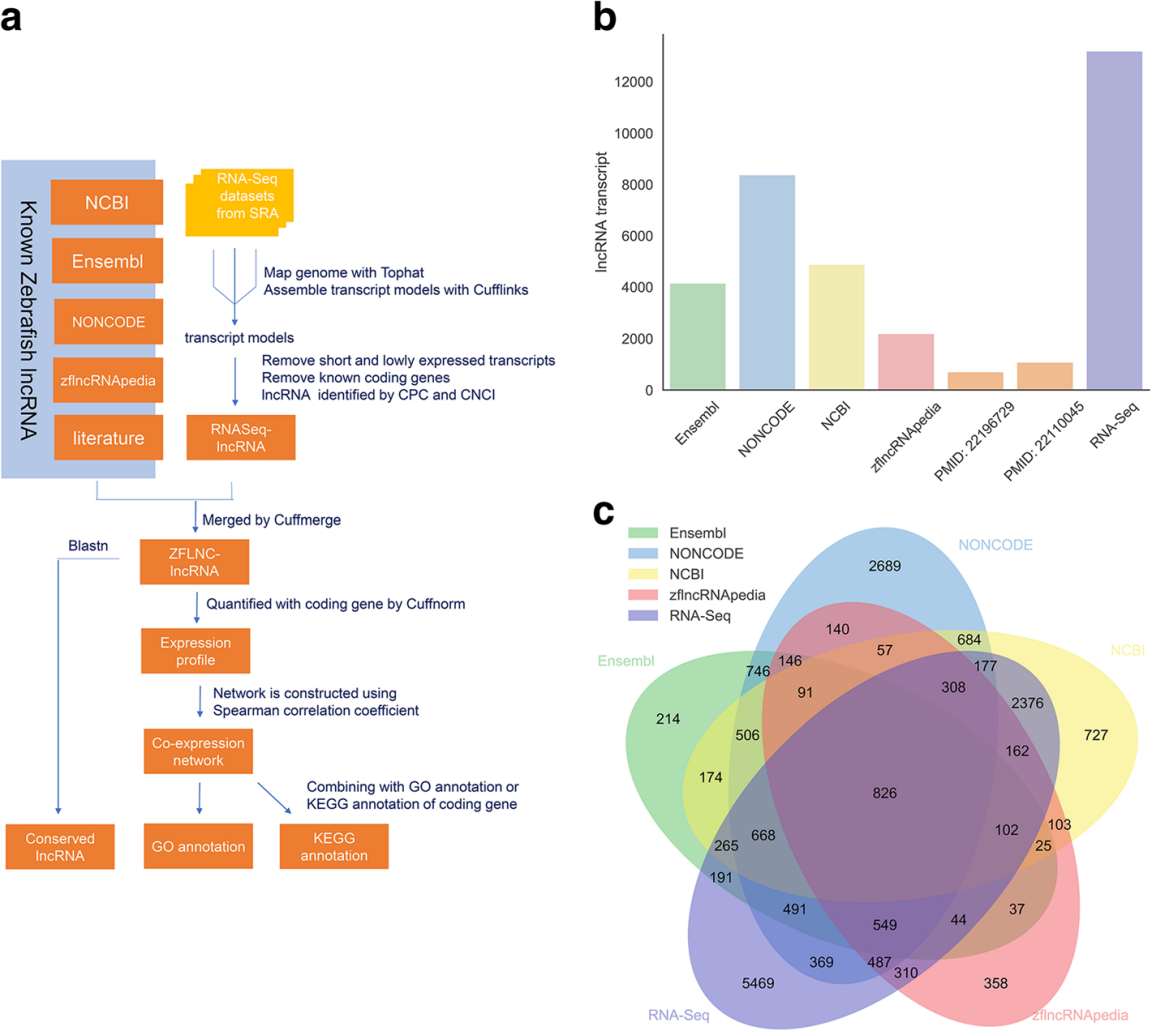
Integration of all sources of zebrafish lncRNA. **a** Data sources and analysis pipeline. **b** The number of lncRNA transcripts from each source. **c** Venn diagram between different sources

To acquire the zebrafish lncRNAs as comprehensive as possible, we made a pipeline for data collection, integration and annotation (Fig. 1a). First, we collected all possible lncRNAs of zebrafish from publicly available databases and literatures, and further integrated them with RNA-seq datasets covering 499 runs in 56 studies from NCBI SRA database (Additional file 1). Using the in-house “RNA-seq data analysis pipeline”, we obtained our final dataset with more zebrafish lncRNAs, containing in total 13,604 lncRNA genes (21,128 transcripts) (Additional file 2). The major data sources of our lncRNA set are from RNA-seq data analysis and NONCODE [22], followed by NCBI, Ensembl and zflncRNApedia orderly [23–25] (Fig. 1b). Venn diagram shows that, although there is an obvious overlapping between different sources, a lot of unique lncRNAs emerged in RNA-seq datasets and NONCODE (Fig. 1c).

**Fig. 2:**
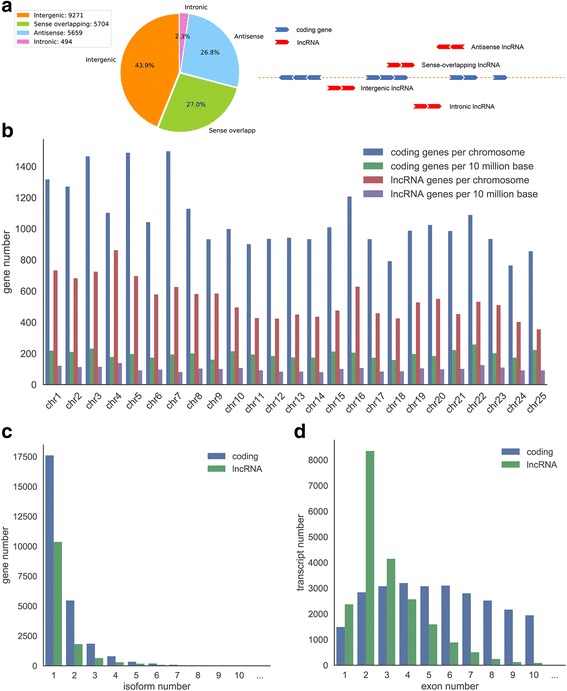
Features of Zebrafish lncRNA. **a** Distribution of lncRNA subtypes. **b** Zebrafish lncRNA distribution in chromosomes. **c** Distribution of zebrafish lncRNA isoform number. **d** Distribution of zebrafish lncRNA exon number

According to the genomic location, these zebrafish lncRNA genes are cataloged as intergenic, sense overlapping, antisense and intronic, in the percentage of 43.9%, 27.0%, 26.8% and 2.3% respectively (Fig. 2a). Similar to coding-gene, zebrafish lncRNA genes have a relatively uniform distribution on chromosome, but less density. There are about 100 lncRNA genes per 10 M sequence, while coding-genes are about 200 genes per 10 M sequence (Fig. 2b). As for the number of isoform for each gene, lncRNA genes are also in line with coding-genes: (1) about 76.1% of lncRNA genes are single-isoform genes, which occupy 66.5% in coding-genes; (2) lncRNA genes with isoform≤3 account to 94.2%, corresponding to 94.1% of coding-genes; (3) lncRNA genes with isoform≥4 only have 5.8% percentage, consistent with 5.9% of coding-genes (Fig. 2c). However, the exon number distribution of zebrafish lncRNAs is far from that of coding-gene, in that the amount of transcripts with multi-exons is significantly less (Fig. 2d). About 40% of zebrafish lncRNA transcripts have two exons, but for coding-genes, the percentage is only 6.7%; in reverse, transcripts with exon number ≥ 5 are increasing in coding-genes (75%), compared with lncRNAs (17.5%).

### Construction of zebrafish coding-lncRNA gene co-expression network

To establish the coding-lncRNA gene co-expression network of zebrafish, we used all collected RNA-seq datasets to quantify the expression levels of both coding- and lncRNA- genes in different tissues and conditions (Additional file 3). The genes with high-expressional variance (top 75 percentile) were selected for identification of co-expressed gene-pairs using Spearman’s correlation coefficient. The *P*-value was estimated by Fisher transformation, and the sets of *P*-value for each gene were adjusted by the Bonferroni method. Only gene-pairs with an adjusted *P*-value of 0.01 or less are retained in subsequent analyses.

**Fig. 3:**
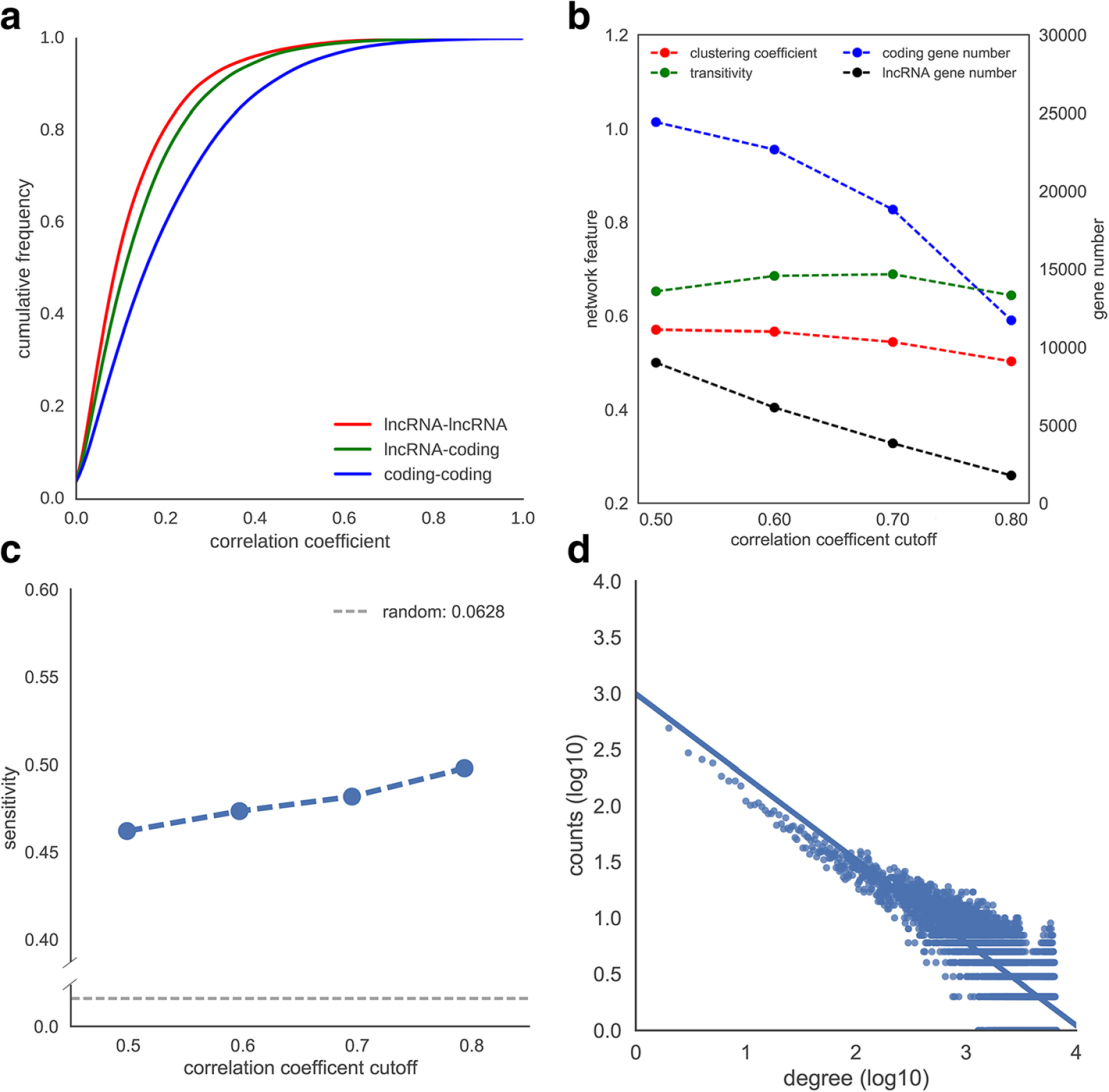
Features of zebrafish coding-lncRNA gene co-expression network. **a** Cumulative distribution of gene expression Spearman’s correlation coefficient. **b** Network statistics by different correlation coefficient cutoffs. **c** Evaluation of function prediction performance of the network with different cutoffs. **d** Network degree distribution (correlation coefficient cutoff = 0.5)

By comparison of the correlation coefficients of gene expression between coding-genes, between lncRNA and coding-gene, and between lncRNAs, respectively (Fig. 3a), we found that the expression correlation between inter-lncRNAs is obviously weaker than inter-coding-genes (Kolmogorov-Smirnov test, *P*-value = 1.45e-232). Intriguingly, the expression correlation between lncRNA and coding-gene is visibly enhanced, as compared with inter-lncRNAs (Kolmogorov-Smirnov test, *P*-value = 3.49e-33), which is likely due to the functional synergia of lncRNAs with their protein partners.

We further evaluated the networks using different cutoffs of Spearman’s correlation coefficient by several network parameters (Fig. 3b). With the ascending of cutoff value, the network size, that is consisting of both coding- and lncRNA- genes, naturally decreased. Yet, the average clustering coefficient and the transitivity scores, two parameters that reflect small-world characters of gene network, still held steady or slightly declined. Moreover, coding-genes having 10 or more known coding neighbors were used as a test set to evaluate the functional prediction performance for the network with different cutoffs (Fig. 3c). We found that, for each cutoff, its prediction performance is higher than randomly selected node; furthermore, the prediction sensitivity increases as the cutoff rises, since more GO annotations of coding-genes can be precisely predicted by their neighbor nodes. This indicated that the higher the cutoff, the more similar the annotated functions of connected gene-pairs in network.

According to the size and quality of these networks, we selected the network that has a Spearman’s correlation coefficient cutoff of 0.5 for follow-up analysis. This coding-lncRNA gene co-expression network shows a scale-free distribution of connectivities (Fig. 3d). There were 9015 non-coding-genes and 24,425 coding-genes that were linked by 25,097,918 edges. Nearly 17,800,940 edges (70.93%) were connected among coding-genes, and 6,253,398 edges (24.92%) were connected among coding- and noncoding- genes, whereas another 1,043,580 edges (4.16%) were linked between pairs of noncoding- genes.

### Functional annotation of zebrafish lncRNAs according to coding-lncRNA gene co-expression network

In our co-expression network established between coding- and lncRNA- gene, the connected gene-pairs tend to have more similar annotated functions, thereby we could use it to further annotate zebrafish lncRNAs’ function, including GO and KEGG pathway. Of the 24,425 coding-genes in this network, 14,650 (59.98%) and 7022 (28.75%) were annotated with at least one GO Biological Process (BP) or KEGG term. Consequently, using functional enrichment analysis by hypergeometric distribution test (*p*-value < 0.05), we annotated each zebrafish lncRNA according to its immediate neighbor coding-genes that have known functional annotation. In this way, we achieved a set of GO (7345 genes) and KEGG (7055 genes) annotation of zebrafish lncRNAs, as listed in Additional file 4.

**Fig. 4:**
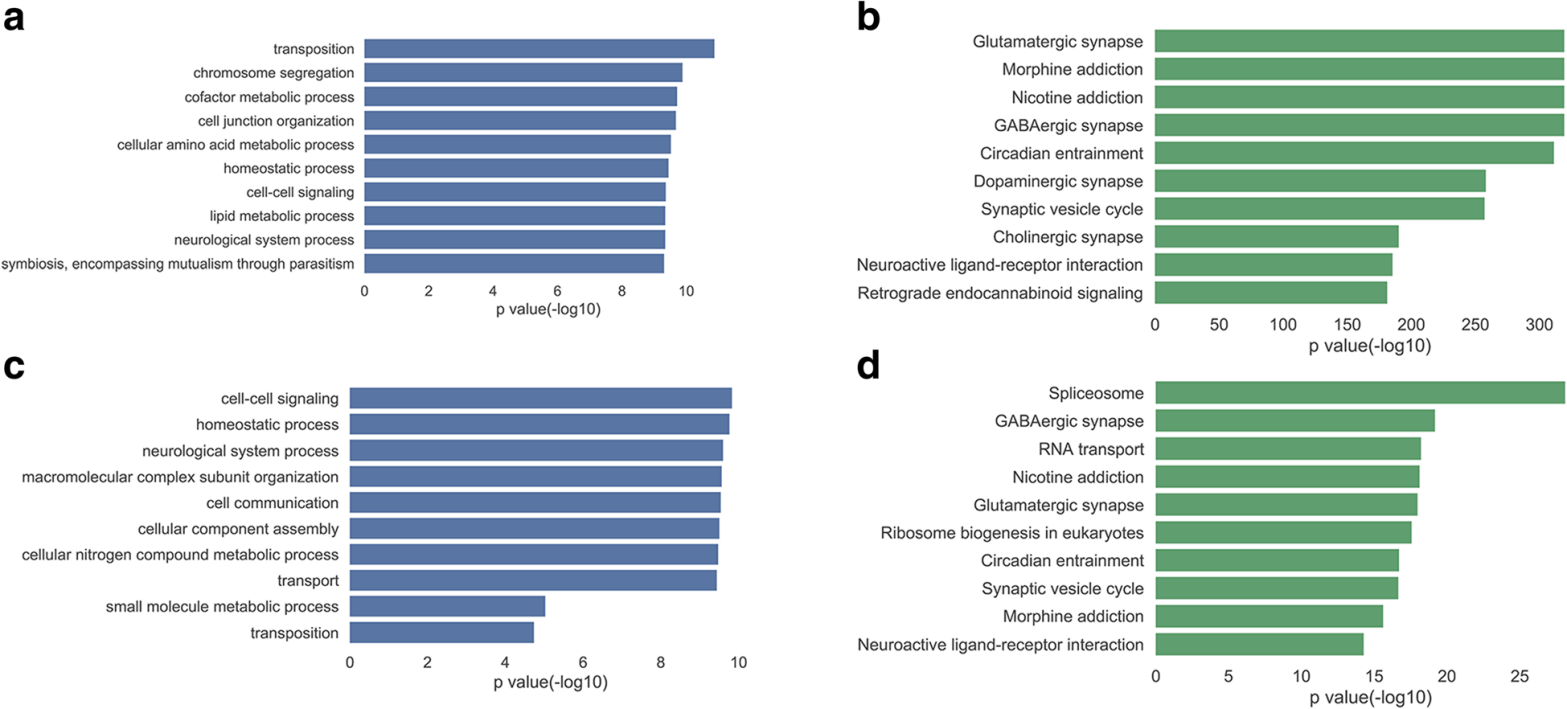
Functional annotation of zebrafish lncRNAs. **a** LncRNA GO BP enrichment slim (top 10). **b** LncRNA KEGG pathway enrichment (top10). **c** Conserved lncRNA GO BP enrichment slim (top 10). **d** Conserved lncRNA KEGG pathway enrichment (top10)

In terms of GO annotation, the top two enriched GO BP terms of zebrafish lncRNAs are associated with chromosome-related processes, as ‘transposition’ (*P*-value = 1.33e-11) and ‘chromosome segregation’ (*P*-value = 1.32e-10) (Fig. 4a). This finding is consistent with previous researches of mammalian lncRNAs, suggesting that lncRNAs can act in the nucleus to regulate the activity of chromosome. For instance, XIST lays over the X chromosome in cis and balances X-linked gene expression [26]; AIR regulates genomic imprinting of a cluster of autosomal genes in cis [27]; else, NORAD controls chromosome segregation and protects cell from becoming aneuploid [28].

In addition, compared with coding-genes, we found that the top ten GO BP terms of zebrafish lncRNAs more tend to be enriched in cell-signaling-related or nervous-system-related processes, such as ‘cell-cell signaling’ (*P*-value = 4.33e-10), ‘cell junction organization’ (*P*-value = 2.1e-10)’, ‘homeostatic process’ (*P*-value = 3.56e-10) and ‘neurological system process’ (*P*-value = 4.5e-10). Interestingly, this finding is intensely echoed by the results of KEGG annotation, in that the top ten KEGG pathways of zebrafish lncRNAs are subtotally correlated to cell signaling and nervous system (Fig. 4b), such as ‘Glutamatergic synapse (*P*-value = 5.9e-320), ‘Morphine addiction’ (*P*-value = 5.9e-320), ‘Neuroactive ligand-receptor interaction’ (*P*-value = 5.02e-186), and ‘Retrograde endocannabinoid signaling’ (*P*-value = 6.2e-182). Since previous studies have demonstrated that mammalian lncRNAs incline to be expressed specifically in the central nervous system and may play central roles in regulation of the development and function of nervous system [29, 30], we reasoned that zebrafish would be very compatible as a research platform for exploring lncRNA function in human nervous system.

### Conservation analysis of zebrafish lncRNAs

For seeking out the putative mammalian orthologs of zebrafish lncRNAs, we used BLASTN to directly compare lncRNAs in zebrafish with that in human or mouse by turns. Those lncRNAs with best hits in bidirectional comparison were considered as lncRNA orthologs (E-value<=10–5). In total, we identified 1828 conserved zebrafish lncRNA genes (1890 transcripts). Therein, 1258 zebrafish lncRNA transcripts have their corresponding orthologs in human; while 1099 zebrafish lncRNA transcripts have mouse orthologs; moreover, 467 zebrafish lncRNA transcripts have the overlapped orthologs in both human and mouse (Additional file 5).

When compared with all lncRNAs, the top ten GO BP terms for conserved zebrafish lncRNA are analogously associated with cell-signaling-related and chromosome-related processes, such as ‘cell-cell signaling’ (*P*-value = 1.49e-10) and ‘transposition’ (*P*-value = 1.84e-05). There are a few of new GO BP terms added, such as ‘macromolecular complex subunit organization’ (*P*-value = 2.73e-10) and ‘cellular component assembly’ (*P*-value = 3.14e-10) (Fig. 4c). Correspondingly, in the top ten KEGG pathways, several novel roles, like ‘Spliceosome’ (*P*-value = 7.91e-29), ‘RNA transport’ (*P*-value = 6.05e-19) and ‘Ribosome biogenesis in eukaryotes’ (*P*-value = 2.7e-18), are present (Fig. 4d). This suggested that the conserved zebrafish lncRNAs are likely implicated in some of key biological processes in nucleus.

**Fig. 5:**
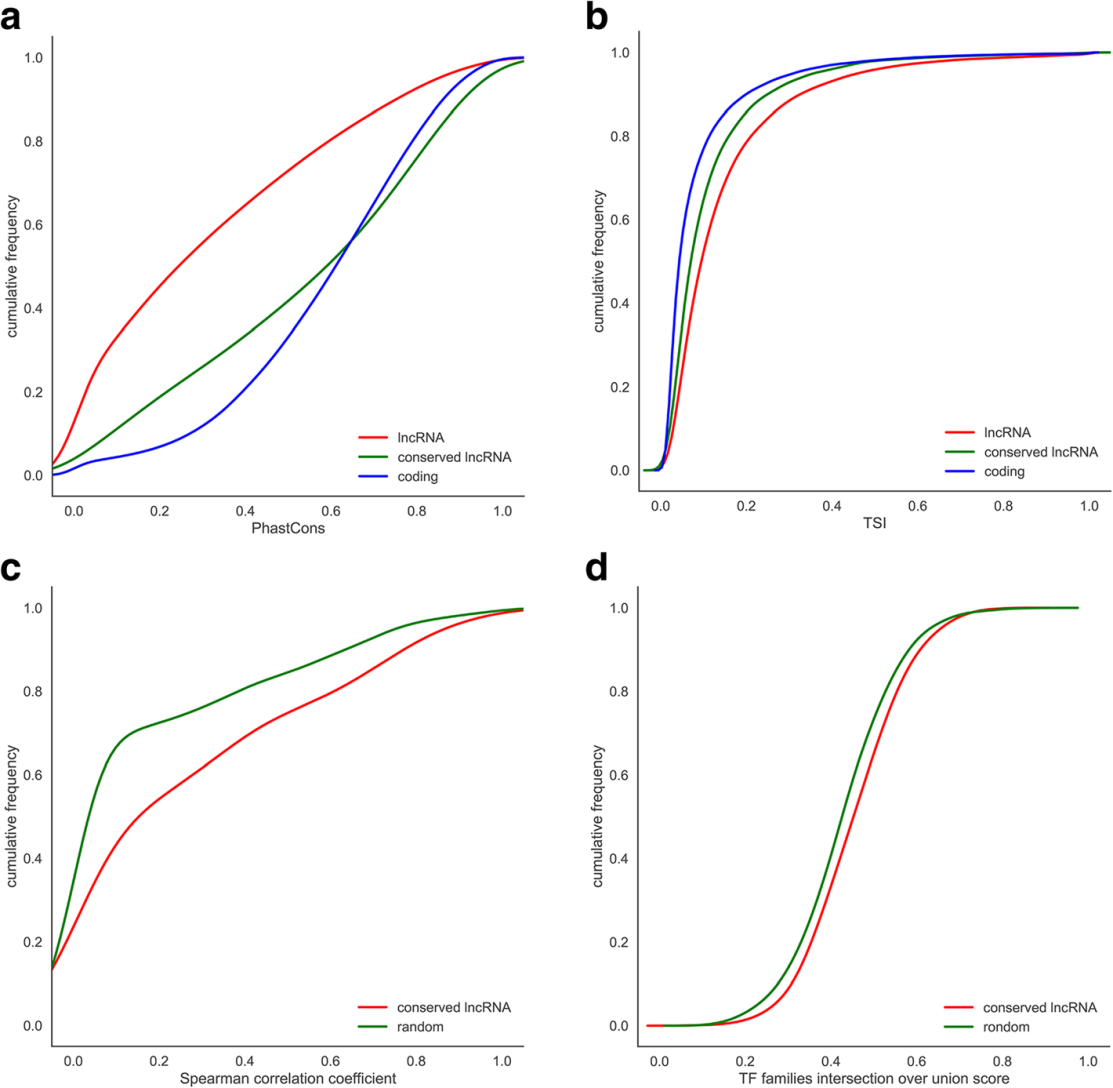
Conservation analysis of zebrafish lncRNAs. **a** Cumulative distribution of conservation levels computed using PhastCons applied to the 8-way whole-genome. **b** Cumulative distribution of TSI (tissue specificity index). **c** Cumulative distribution of Spearman’s correlation coefficient of gene expression. **d** Cumulative distribution of TF families’ intersection over union score

We also evaluated the full-transcript conservation levels of those conserved zebrafish lncRNAs using 8-way PhastCons scores (Fig. 5a). It was found that the PhastCons scores of conserved zebrafish lncRNA are close to that of zebrafish coding-gene, both of which are significantly greater than the entirety of all zebrafish lncRNAs (Kolmogorov-Smirnov test, *P*-value = 6.35e-155). Then, we examined the tissue expression specificity against diverse tissues and different conditions, amongst zebrafish lncRNA, conserved lncRNA and coding-gene. Unlike the unconserved ones, conserved lncRNAs are more resemblance of mRNA as displaying a less tissue-specific expression pattern (Kolmogorov-Smirnov test, *P*-value = 1.58e-12), thereby may express and execute functions in more extensive tissues (Fig. 5b).

Furthermore, we investigated the multi-tissue expression profiles of conserved zebrafish lncRNAs, as compared with their mammalian counterparts (Fig. 5c). The conserved zebrafish lncRNAs, along with their mammalian orthologs, have a more strengthened correlation in tissue-expression profiles at heart, liver, muscle, brain and blood tissues, in comparison of random lncRNA-pairs between zebrafish and human (Kolmogorov-Smirnov test, *P*-value = 1.44e-07). We have also evaluated the number of upstream transcription factor (TF) families shared by both conserved zebrafish lncRNA and its human counterpart (Fig. 5d). It was found that the conserved lncRNAs have a significant conformity with their mammalian counterparts in upstream regulatory pattern (Kolmogorov-Smirnov test, *P*-value = 1.95e-3). Our results indicated that, as compared with their mammalian counterparts, the conservative property of conserved zebrafish lncRNAs reflects not only on gene sequence, probably also on the aspects of tissue expression and transcription regulation pattern.

### ZFLNCG05544 is a candidate lncRNA gene related to human neuron diseases

Based on our own results on GO and KEGG annotation, multi-tissue expression profile and coding-lncRNA gene co-expression network, we made an attempt to explore the potential function of zebrafish lncRNA. Previous studies showed that a considerable amount of mammalian lncRNAs are specifically expressed in central nervous system, and thus may play important roles in nervous system development and maintenance [29, 30]. Likewise, we discovered that there are 2423 zebrafish lncRNAs related to neuron, according to their GO and/or KEGG annotation. After confining the tissue type and the expression level of zebrafish lncRNAs (Brain, average FPKM> = 10), this number narrows down to 467. Further, we examined the co-expression state between the latter and known human-neuron-disease-related coding-genes in zebrafish (data collected from ZFIN, http://zfin.org) [13]. Consequently, one lncRNA gene, ZFLNCG05544, attracted our attention.

**Fig. 6:**
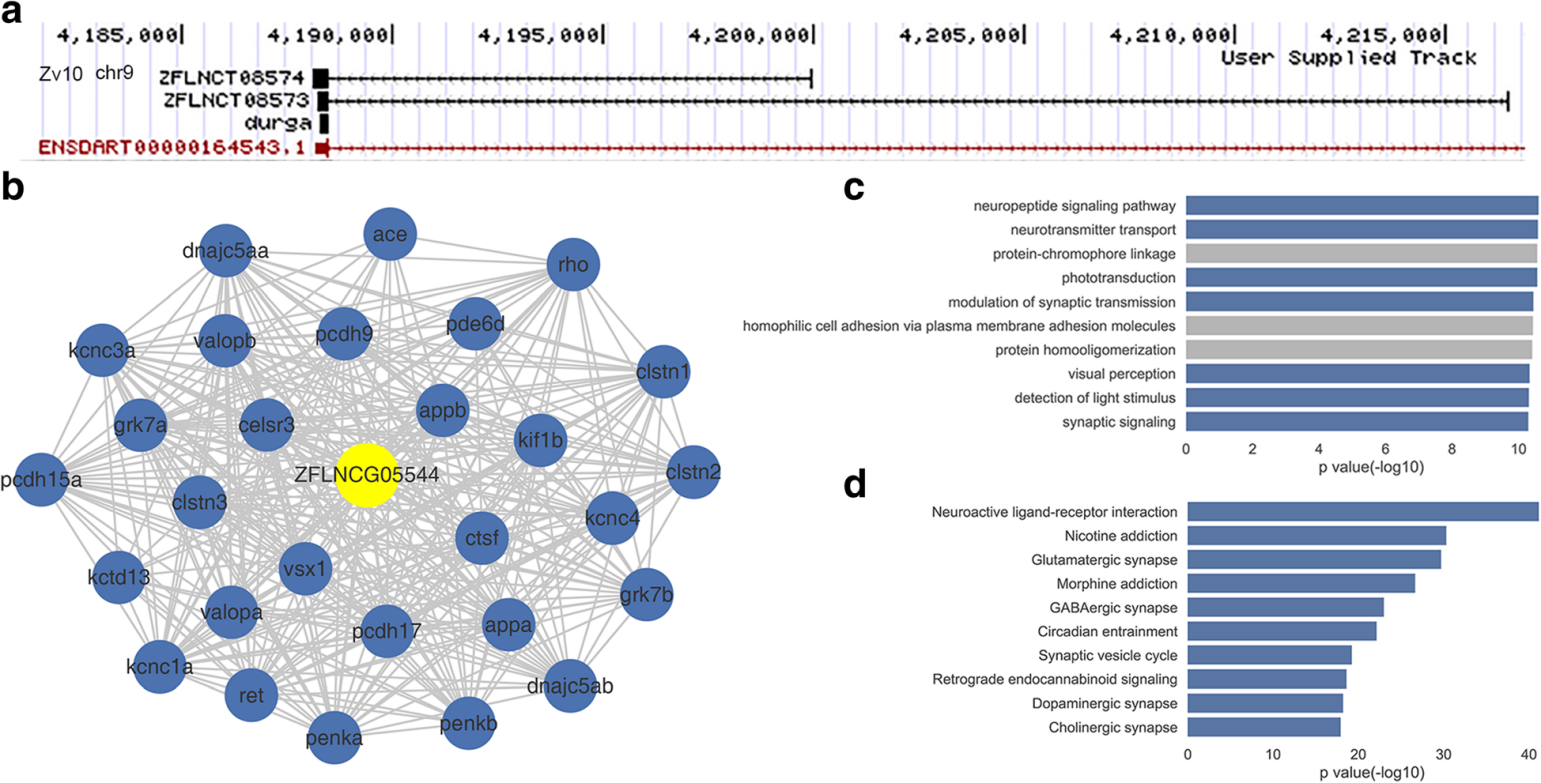
ZFLNCG05544 is a candidate lncRNA gene related to human neuron diseases. **a** The two transcripts of ZFLNCG05544(ZFLNCT08573, ZFLNCT08573) and durga in UCSC genome browser. **b** ZFLNCG05544 co-expression subnetwork. **c** GO annotation of ZFLNCG05544 (top10). **d** KEGG annotation of ZFLNCG05544 (top10)

ZFLNCG05544 locates in chromosome 9, containing two transcripts ZFLNCT08573 and ZFLNCT08574 with the length of 288 nt and 402 nt respectively (Fig. 6a). ZFLNCG05544 is highly expressed in Brain (average FPKM = 27.11, look the details in Additional file 3). The top ten GO annotations of ZFLNCG05544 tend to be enriched in neuron-related processes, such as ‘neuropeptide signaling pathway’ (*P*-value = 2.5e-11), ‘neurotransmitter transport’ (*P*-value = 2.61e-11), ‘modulation of synaptic transmission’ (*P*-value = 3.59e-11), and ‘synaptic signaling’ (*P*-value = 5.12e-11) (Fig. 6c). Equally, the top ten KEGG annotations of ZFLNCG05544 are also linked to neuron, such as ‘Neuroactive ligand-receptor interaction’ (*P*-value = 7.3e-42), ‘Nicotine addiction’ (*P*-value = 4.9e-31), ‘Glutamatergic synapse’ (*P*-value = 2.0e-30), ‘Circadian entrainment’ (*P*-value = 7.4e-23), and ‘Retrograde endocannabinoid signaling’ (*P*-value = 2.5e-19) (Fig. 6d).

As shown by the co-expression sub-network centered around ZFLNCG05544, there is a concurrent expression of ZFLNCG05544 with many neuron-related coding-genes, especially those relevant to known human neuron diseases, like ace, appa, appb, ctsf, dnajc5aa, dnajc5ab, kif1b and tfg (Fig. 6b). For example, the amyloid precursor protein (APP) has been a focus of intense investigation because of its association with Alzheimer’s disease [31]. Previous studies have shown that the zebrafish APP homologue, appb, is required for motor neuron guidance and synapse formation, and is essential for the formation of Mauthner cell in the hindbrain during development [32, 33]. Another example is kinesin family member 1B (kif1b), which has been associated with susceptibility to multiple sclerosis. Lyons et al. have found that kif1b is essential for mRNA localization in oligodendrocytes and development of myelinated axons in zebrafish [34]. ZFLNCG05544 is intensely linked to these neruron-related coding-genes, as indicated by our coding-lncRNA gene co-expression network, implying its important roles in nervous system development and maintenance.

A very recent study gave a powerful support to our prediction of ZFLNCG05544 functions. Sarangdhar et al. demonstrated that zebrafish lncRNA durga modulates dendrite density through regulation of expression of kalrna, which is a coding-gene playing a key role in axonal development, nerve growth and synaptic re-modeling [35]. Therein, lncRNA durga locates in the first exon of kalrna in an antisense orientation, and belongs to the catalogue of antisense lncRNA. By direct genomic mapping and BLAST alignment, we found that ZFLNCG05544 has the same location and strand direction in genome, when compared with lncRNA durga, but with a slight discrepancy in transcript length. Thus, they are probably different transcripts for the same gene. (Fig.6a). In view of the kalrna’s association with many pathological conditions like schizophrenia and autism-spectrum disorders [36, 37], we thought that ZFLNCG05544 could be a proper candidate lncRNA gene related to multifarious human neuron diseases.

### ZFLNCG08251 is a human MALAT1 homolog in zebrafish

Herein, we seek out 1890 conserved zebrafish lncRNAs, which have the corresponding mammalian orthologs. By further comparing them with those known functional human lncRNAs (101 entries in total) collected in lncRNAdb [38], twelve of conserved zebrafish lncRNAs in our own analysis hit their well-characterized human orthologs, including 7SK, 7SL, BC200, CCAT1, JPX, KRASP1, MALAT1, MASCRNA, MEGAMIND, OTX2OS1, PTENP1, RAB4B-EGLN2. Amongst them, MALAT1 can parallel a lncRNA ZFLNCG08251 in zebrafish chromosome 14, a transcript of 7540 nt length that is proximal to MALAT1 (8545 nt) (Additional file 2). This result is consistent with the discovery of Ulitsky et.al [20], in that a lncRNA transcript, in the same place of ZFLNCG08251, had been annotated to relevance of human and mouse’s MATLAT1, yet fail to further function identification.

**Fig. 7:**
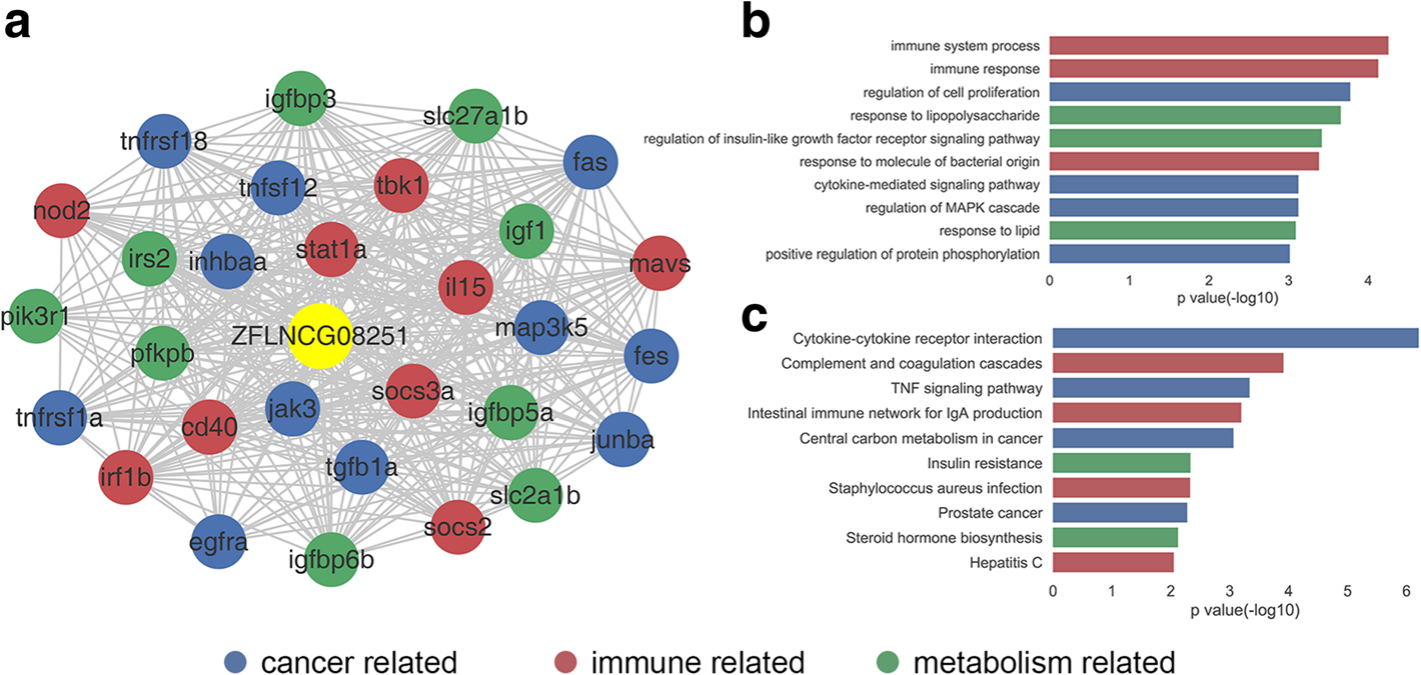
ZFLNCG08251 is a human MALAT1 homolog in zebrafish. **a** ZFLNCG08251 co-expression subnetwork. **b**. GO annotation of ZFLNCG08251 (top10). **c** KEGG annotation of ZFLNCG08251 (top10)

MALAT1 is a large, infrequently spliced non-coding RNA, which was closely related to various pathological processes, such as cancer, diabetes complications, and innate immunity [39–42]. Excitingly, the top ten GO annotations of ZFLNCG08251 are enriched in the exactly same categories of cancer, metabolism and immune (Fig. 7b), such as ‘regulation of cell proliferation’ (*P*-value = 1.7e-04), ‘regulation of insulin-like growth factor receptor signaling pathway’ (P-value = 3.8e-04), and ‘immune system process’ (P-value = 5.6e-05). Moreover, its corresponding top ten KEGG pathways are also encapsulated within cancer, metabolism, immune annotation (Fig. 7c), such as ‘Central carbon metabolism in cancer’ (*P*-value = 8.7e-04), ‘Insulin resistance’ (*P*-value = 4.6e-03), and ‘Intestinal immune network for IgA production’ (*P*-value = 6. 4e-04).

In addition, the co-expression sub-network of ZFLNCG08251 shows that it is likely to actively involve in the pathological processes of cancer, diabetes complications, and innate immunity etc., as its human counterpart (Fig. 7a). Amongst the neighbor genes co-expressed with ZFLNCG08251, we detected extensively three types of genes, which are correlated to cancerous signaling pathway (e.g. fas, fes, egfra, map3k5, junba, tgfb1a, tnfsf12, tnfrsf1a, tnfrsf18, jak3, inhbaa), immunity (e.g. il15, irf1b, cd40, tbk1, mavs, socs2, socs3a, stat1a, nod2), and glycometabolism (e.g. insulin-like growth factor-binding protein -- igfbp3, igfbp5a, igfbp6b, insulin-like growth factor -- igf1, insulin receptor substrate -- irs2). All these results hint that ZFLNCG08251, along with its human homolog MALAT1, not only has the sequence conservation, but also the conservative function.

## Discussion

Zebrafish is recognized as one of the most important vertebrate model organisms, based on the fact that the development and function of zebrafish organs are strikingly similar to human. Combined with the ease of creating mutant or transgenic individual, zebrafish has been served as an important model system for studying human-disease-related protein-coding genes [14–16]. Nevertheless, the comparable study on zebrafish lncRNAs is still scanty, resulting in part from the deficiency of systematic collection and characterization of lncRNAs, as well as the incomplete quantification of their expression profiles. On the other hand, the lack of systematic annotations of zebrafish lncRNAs’ function and conservation also made a big obstacle to the follow-up study.

In this work, we provided by far the most comprehensive dataset of zebrafish lncRNAs, which is consist of multiple information of zebrafish lncRNAs, like expression profile, co-expression network, function and conservation annotation etc. Our functional analysis uncovered that, similar to mammalian lncRNA, zebrafish lncRNAs also tend to play roles in regulation of the development and function of nervous system; additionally, conservation analysis indicated that the conserved zebrafish lncRNAs have both sequential and functional conservative with their mammalian counterparts. Therefore, we inferred that zebrafish would be an applicable platform for exploring lncRNA function in development processes and human diseases.

Unlike the protein-coding gene, it is very difficult to directly annotate lncRNA’s functions by sequence analysis, due to the lack of information about lncRNA functional and/or structural domain. The existing studies had revealed that mammalian lncRNAs often execute functions with their protein partners, this fact is verified by our analysis of coding-lncRNA gene co-expression in zebrafish. On account of this, the annotation for lncRNA often utilizes the relevant co-expressed coding-genes in network as to enrich lncRNA’s function [43, 44]. Compared with human and mouse, the high-throughput sequencing data of zebrafish lncRNA transcriptome are still in less scale. We believed that, in the future, accumulating data of zebrafish transcriptome would further strength our power of lncRNA function prediction based on co-expression network.

Zebrafish shares a high genetic similarity to human, and lots of known human-disease-related coding-genes have functional homologs in zebrafish. However, the conservative of zebrafish lncRNAs is worthy to further gain insight [45]. Through direct BLASTN search, we found 1890 conserved zebrafish lncRNAs, accounting for about 8.9% of total zebrafish lncRNAs (1890/21,128), this result is consistent with the discovery on conservation analysis by Ulitsky et.al (5.3%, 29/550) [20]. In view of the low conservation of lncRNA sequence, syntenic conservation analysis will facilitate to search out the homologs of less conserved lncRNAs, other than the traditional direct BLASTN search [46]. Besides the increased sensitivity of conservation analysis, how to ensure the analysis’ specificity is still an open question.

## Conclusions

In summary, by integrative data analysis and construction of coding-lncRNA gene co-expression network, we gained by far the most comprehensive dataset of zebrafish lncRNAs, as well as their systematic annotations and comprehensive analyses on function and conservation. Our study provides some important insights into the functional roles of zebrafish lncRNAs and their possible application for studying human diseases. We anticipate that this study will provide a reliable zebrafish-based platform to deeply explore lncRNA function and mechanism, and make a roadmap to investigate the commonality of lncRNAs’ function and conservation between zebrafish and human.

## Methods

### Data sources

RNA-Seq data are downloaded from NCBI SRA database; known zebrafish lncRNAs are collected from NCBI [24], Ensembl [25], NONCODEv4 [22], zflncRNApedia [23] and literatures [20, 21] (See Additional file 1 for details).

### RNA-seq data analysis pipeline

SRA format files were dumped to FASTQ format files by SRA-Toolkit. Low quality reads were trimmed by Trimmomatic (Version 0.32) [47]. RNA-Seq reads were mapped to zebrafish genome (Zv9) using Tophat2 (Version 2.0.13) [48], then transcriptome was assembled by Cufflinks (Version 2.2.1) [49]. Multiple-exon transcripts were considered as being expressed if they had an FPKM greater than 0.1. For single-exon transcript, more rigorously, FPKM should be greater than 5 and transcript length greater than 2000. Those foregone coding-genes or transcripts with size less than 200 nt were filtered out. Then, lncRNA candidates were identified by CPC (Version 0.9-r2) [50] and CNCI (Version 2) [51]. At last, all zebrafish lncRNAs stemming from RNA-seq datasets and other publicly available sources were integrated together using the Cuffmerge program in the Cufflinks suite.

### Coding-lncRNA gene co-expression network construction

The expression profile of zebrafish lncRNAs and coding-genes were quantified by Cuffnorm program in the Cufflinks suite and then scaled by upper-quartile normalization (Eq. 1). We then calculated the Spearman’s correlation coefficient and its corresponding *P*-value (Eq. 2) between the expression profiles of each gene-pair using the in-house Perl script. Only gene-pair with an adjusted P-value of 0.01 or less and with a Spearman’s correlation coefficient no less than 0.5 is regarded as co-expression in our coding-lncRNA gene co-expression network.

1 $$ {\rho}_i=\frac{\frac{1}{n}{\sum}_i^n{k}_i}{k_i} $$

For each sample i, *k*_*i*_ is its upper quartile of all gene expression values different from 0, n is the number of all samples, *ρ*_*i*_ is the scaling factor for sample i. The upper quartile normalization for all gene expression values in sample i is obtained by multiplying them to the scaling factor *ρ*_*i*_.

2 $$ \left\{\begin{array}{c}\mathrm{Rs}=\frac{\sum_i\left({x}_i-\overline{x}\right)\left({y}_i-\overline{y}\right)}{\sqrt{\sum_i{\left({x}_i-\overline{x}\right)}^2{\sum}_i{\left({y}_i-\overline{y}\right)}^2}}\\ {}\mathrm{F}\left(\mathrm{Rs}\right)=\frac{1}{2}\ln \frac{1+\mathrm{Rs}}{1-\mathrm{Rs}}\kern3.5em \\ {}\mathrm{Z}=\sqrt{\frac{\mathrm{n}-3}{1.06}}\mathrm{F}\left(\mathrm{Rs}\right)\kern4.25em \end{array}\right. $$

Where x or y represents the vector of the ranked expression value of each gene, Rs is the Spearman’s correlation coefficient between x and y, *x*_*i*_ or *y*_*i*_ stands for the rank of each expression value, $\overline{x}$ or $\overline{y}$, is the mean value of these ranks. F (Rs) is the Fisher transformation of Rs, and n is the sample size i.e. the vector length. The corresponding *P*-value of each Rs is calculated from Z, which is a z-score for Rs that approximately follows a standard normal distribution under the null hypothesis of statistical independence [52, 53].

### Network statistics

The clustering coefficient score C for a graph is the average of local clustering coefficient values C_i_ of each node (Eq. 3). The transitivity T of a graph is based on the relative number of triangles in the graph, compared to total number of connected triples of nodes (Eq. 4).

3 $$ \left\{\begin{array}{c}{\mathrm{C}}_{\mathrm{i}}=\frac{number\ of\ triangles\ connected\ to\ node\ i}{number\ of\ triples\ centered\ around\ node\ i}\\ {}C=\frac{1}{\mathrm{n}}{\sum}_{\mathrm{i}=1}^{\mathrm{n}}{\mathrm{C}}_{\mathrm{i}}\end{array}\right. $$

Where If node i has two neighbor nodes and these two nodes are also connected, there is a triangle connected to node i, a triple centered around node i is a set of two edges connected to node i, n is the number of node in the network. By definition, 0 ≤ *C*_*i*_ ≤ 1 and 0 ≤ C ≤ 1.

4 $$ T=\frac{3\times number\ of\ triangles\ in\ the\ network}{number\ of\ connected\ triples\ of\ nodes\ in\ the\ network} $$

The factor of “3” in number accounts for the fact that each triangle contributes to three different connected triples in the graph, one centered at each node of the triangle. With this definition, 0 ≤ *T* ≤ 1, and *T* = 1 if the network contains all possible edges.

### LncRNA functional annotation

The GO annotation of zebrafish coding-gene was downloaded from Gene Ontology Consortium (only biological process annotations were considered). While, GO annotation of zebrafish lncRNA was predicted using the goatools (version 0.6.4) [54], which determines the GO annotation of one gene in our network according to the GO annotations of its immediate neighbor genes (*P*-value < 0.05).

The KEGG annotation of zebrafish coding-gene was obtained from KEGG Automatic Annotation Server using zebrafish coding-gene sequence [55]. While, KEGG annotation of zebrafish lncRNA was predicted using the in-house Python script. The KEGG annotation of one gene in our network was determined by the enrichment of KEGG annotations according to its immediate neighborhood (*p*-value < 0.05), when using hypergeometric distribution (Eq. 5).

5 $$ \mathrm{P}=1-{\sum}_{i=0}^{k-1}\frac{\left(\genfrac{}{}{0pt}{}{M}{i}\right)\left(\genfrac{}{}{0pt}{}{N-M}{n-i}\right)}{\left(\genfrac{}{}{0pt}{}{N}{n}\right)} $$

In this equation, N is the total number of genes in the network, M is the total number of genes having one certain KEGG annotation, n is the number of a gene’s immediate neighbors and k is the number of neighbor genes having one certain KEGG annotation.

### Analysis of conserved lncRNAs

Zebrafish lncRNAs’ putative mammalian orthologs were inferred from bidirectional best hits in direct comparison of zebrafish lncRNA and human lncRNA or mouse lncRNA with BLASTN using a relatively non-stringent E-value threshold 10^− 5^. Zebrafish lncRNAs’ conservation levels were evaluated using 8-way PhastCons score [56].

Using expression profiles of all SRA runs, the tissue specificity of zebrafish lncRNAs’ gene expression was calculated by TSI (Eq. 6).

6 $$ \mathrm{TSI}=\frac{\max \left({\mathrm{x}}_{\mathrm{i}}\right)}{\sum \limits_{\mathrm{i}}{\mathrm{x}}_{\mathrm{i}}} $$

Where TSI is the tissue specificity index, x_i_ is the expression value.

To obtain the correlation of tissue expression among conserved lncRNAs, Spearman’s correlation coefficient scores were calculated for each conserved zebrafish lncRNA and its human counterpart. The tissue-expression profile data of zebrafish come from five organs, including heart, liver, muscle, brain and blood, as reported by Kaushik et.al. [57]; while, that of human from Human body map.

To estimate the conformity of upstream (from − 500 to + 100) regulatory patterns of conserved lncRNAs, we evaluated the number of upstream transcription factor (TF) families shared by both conserved zebrafish lncRNA and its human counterpart using intersection over union score (Eq. 7). TF-binding site prediction was performed using the HOMER tool [58].

7 $$ \mathrm{S}\kern0.5em =\kern0.5em \frac{\cap \left({\mathrm{TF}}_{\mathrm{Z}},{\mathrm{TF}}_{\mathrm{H}}\right)}{\cup \left({\mathrm{TF}}_{\mathrm{Z}},{\mathrm{TF}}_{\mathrm{H}}\right)} $$

Where S is the intersection over union score of upstream TF families shared by both conserved zebrafish lncRNA (TF_Z_) and its human counterpart (TF_H_).

## Additional files

Additional file 1: Data source. (XLSX 33 kb)

Additional file 2: lncRNA dataset. (ZIP 10705 kb)

Additional file 3: lncRNA-coding expression profiles. Replicate samples are merged. (ZIP 13305 kb)

Additional file 4: lncRNA GO and KEGG annotation. (ZIP 17127 kb)

Additional file 5: Conserved lncRNA. (XLSX 84 kb)

## Abbreviations

BP: Biological Process
FPKM: Fragments Per Kilobase Million
GO: Gene Ontology
KEGG: Kyoto Encyclopedia of Genes and Genomes
LncRNA: Long non-coding RNA
TF: Transcription Factor
TSI: Tissue Specificity Index
